# Psychometric validation of the Dutch translation of the quality of life in reflux and dyspepsia (QOLRAD) questionnaire in patients with gastroesophageal reflux disease

**DOI:** 10.1186/1477-7525-8-85

**Published:** 2010-08-17

**Authors:** Leopold GJB Engels, Elly C Klinkenberg-Knol, Jonas Carlsson, Katarina Halling

**Affiliations:** 1 Department of Gastroenterology, Maasland Hospital, Sittard, the Netherlands; 2 Department of Gastroenterology, VU Medical Centre, Amsterdam, the Netherlands; 3 Outcomes Research, AstraZeneca R&D, 431 83 Mölndal, Sweden; 4 PRO consulting, Stora Åvägen 21, 436 34 Askim, Sweden; 5 Affiliation at the time the study was conducted

## Abstract

**Background:**

The Quality of Life in Reflux and Dyspepsia (QOLRAD) questionnaire is one of the best-characterized disease-specific instruments that captures health-related problems and symptom-patterns in patients with gastroesophageal reflux disease (GERD). This paper reports the psychometric validation of a Dutch translation of the QOLRAD questionnaire in gastroenterology outpatients with GERD.

**Methods:**

Patients completed the QOLRAD questionnaire at visit 1 (baseline), visit 2 (after 2, 4 or 8 weeks of acute treatment with esomeprazole 40 mg once daily), and visit 4 (after 6 months with on-demand esomeprazole 40 mg once daily or continuous esomeprazole 20 mg once daily). Symptoms were assessed at each visit, and patient satisfaction was assessed at visits 2 and 4.

**Results:**

Of the 1166 patients entered in the study, 97.3% had moderate or severe heartburn and 55.5% had moderate or severe regurgitation at baseline. At visit 2, symptoms of heartburn and regurgitation were mild or absent in 96.7% and 97.7%, respectively, and 95.3% of patients reported being satisfied with the treatment. The internal consistency and reliability of the QOLRAD questionnaire (range: 0.83-0.92) supported construct validity. Convergent validity was moderate to low. Known-groups validity was confirmed by a negative correlation between the QOLRAD score and clinician-assessed severity of GERD symptoms. Effect sizes (1.15-1.93) and standardized response means (1.17-1.86) showed good responsiveness to change. GERD symptoms had a negative impact on patients' lives.

**Conclusions:**

The psychometric characteristics of the Dutch translation of the QOLRAD questionnaire were found to be satisfactory, with good reliability and responsiveness to change, although convergent validity was at best moderate.

## Background

Gastroesophageal reflux disease (GERD) is a condition that develops when the reflux of stomach contents causes troublesome symptoms and/or complications [1]. The characteristic symptoms of GERD are heartburn and regurgitation, which have a prevalence of 75-98% and 48-91%, respectively, in patients with reflux disease [1]. Dysphagia is also common, especially in individuals with reflux esophagitis [2]. GERD affects many aspects of day-to-day functioning, including sleep, productivity at work and at home, and enjoyment of meals and social occasions [3-5]. Symptoms can also cause emotional distress.

Assessing the impact of reflux symptoms on patients' lives can provide important information on health status and perceived treatment efficacy. Such assessment should be carried out using validated patient-reported outcome instruments. In its draft guidance, the US Food and Drug Administration (FDA) encourages the development of instruments that are able to translate a change in symptoms into specific endpoints such as improvements in the ability to perform daily activities or improvements in psychological state [6]. The FDA evaluates such instruments by their ability to measure specific concepts in a reliable and valid way. It also stipulates that each instrument needs to be specific to the intended population and to the characteristics of the condition or disease treated.

Generic instruments capture a wide range of health-related problems and allow for comparisons across different diseases. In contrast, disease-specific instruments capture health-related problems and symptom patterns that are of particular relevance to a specific condition [7,8]. Disease-specific instruments are generally more responsive than generic instruments in detecting small changes over time, and are thus better suited as outcome measures in interventional studies [7,8].

One of the best-characterized disease-specific instruments for patients with GERD is the Quality of Life in Reflux and Dyspepsia (QOLRAD) questionnaire [9]. The QOLRAD questionnaire measures the impact of reflux symptoms on patients' emotional health, sleep, vitality, eating and drinking, and physical and social functioning. The QOLRAD questionnaire was originally developed in US English, and has subsequently been translated and culturally adapted for use in international studies [10-12]. This paper reports the psychometric validation of a Dutch translation of the QOLRAD questionnaire in patients with GERD.

## Methods

### Patients

Patients with GERD were selected in gastroenterology outpatient clinics. Inclusion criteria required a history of heartburn of at least 3 months, and episodes of heartburn of at least moderate severity for 3 days or more during the 7 days prior to the study. Heartburn was defined as a burning feeling, rising from the stomach or lower part of the chest up towards the neck. The following exclusion criteria were applied: the presence of reflux esophagitis grade C or D, presence or history of other gastrointestinal diseases and conditions, and presence or history of other non-gastrointestinal serious diseases and conditions. Patients treated with proton pump inhibitors or prokinetic drugs during the 14 days preceding endoscopy or who had been treated with non-steroidal anti-inflammatory drugs or *Helicobacter pylori *eradication therapy were also excluded.

Patients received acute treatment for their symptoms with esomeprazole 40 mg once daily for 2, 4 or 8 weeks. The length of acute treatment was dependent on the length of time taken to achieve sufficient symptom relief and patient satisfaction. Patients satisfied with the treatment and with sufficient symptom relief entered the maintenance phase and were randomized to receive on-demand esomeprazole 40 mg once a day or continuous esomeprazole 20 mg once daily for 6 months. Data are presented from visit 1 (baseline), visit 2 (after 2, 4 or 8 weeks of acute treatment with esomeprazole 40 mg), and visit 4 (after 6 months of maintenance treatment) [13].

The study was performed in accordance with the ethical principles of the Declaration of Helsinki, the Good Clinical Practice and the Wet Medisch-Wetenschappelijk Onderzoek met mensen (WMO). The final study protocol, including the final version of the Patient Information and Consent Forms, were approved in accordance with the WMO by an Independent Ethics Committee belonging to the Maasland Hospital, Sittard, the Netherlands.

### Symptom assessment

Investigators recorded patient demographics (including sex, age, height and weight), medical history (including history of reflux symptoms), and drugs used during the month before enrolment. Patients completed the QOLRAD questionnaire at each visit. All patients who prematurely discontinued the study were encouraged to complete the QOLRAD questionnaire at their last visit to the clinic.

At each visit, investigators assessed the severity of patients' heartburn, regurgitation and dysphagia in the 7 days prior to the visit. Symptoms were scored as follows: none (no complaints), mild (aware of symptom, but easily tolerated), moderate (discomforting symptom, sufficient to cause interference with normal daily activities and/or sleep), severe (incapacitating symptom, with inability to perform normal daily activities and/or sleep). Patients completed a daily paper diary during the study treatment period, in which they recorded heartburn severity during the past 24 hours. Patient satisfaction was evaluated at visit 2 and visit 4, using a 4-point Likert scale (completely satisfied, quite satisfied, quite dissatisfied, completely dissatisfied).

### QOLRAD questionnaire

The heartburn version of the QOLRAD questionnaire is a disease-specific quality of life instrument that includes 25 items combined into five domains: Emotional distress, Sleep disturbance, Food/drink problems, Physical/social functioning and Vitality. Questions are rated on a 7-point Likert scale; the lower the value the more severe the impact on daily functioning [9]. Previous studies have shown that a difference of approximately 0.5 points represents a clinically relevant change [4,10]. The QOLRAD questionnaire has been validated in Australia, Canada (French- and English-speaking regions), USA, UK, Germany, Italy, Spain, Hungary, Poland and South Africa [9-12,14,15]. The Dutch version of the QOLRAD questionnaire was developed from the English version by forward-back translation.

### Psychometric evaluation

#### Reliability

Internal consistency refers to the extent to which the items within each domain are interrelated. Cronbach's α coefficient is the most widely used method of assessing internal consistency; a high α coefficient (≥ 0.70) suggests good internal consistency and reliability [16].

Ceiling effects (the proportion of patients having the maximum score) were also assessed. The presence of ceiling effects, in which a high proportion of the patients grade themselves as having the maximum score, indicates that the scales will have poor discrimination. Thus sensitivity and responsiveness is reduced.

#### Construct validity

Construct validity assesses whether an indicator actually measures its underlying attribute. The construct validity was examined by convergent and known-groups validity.

Convergent validity demonstrates whether a postulated instrument domain correlates appreciably with all other domains that should be related to it. Pearson's product moment correlation was used to compare the results of the QOLRAD questionnaire with clinician assessments of reflux symptoms. Similar domains in these instruments were expected to have high correlations with each other. A strong correlation was considered to be over 0.60, a moderate correlation between 0.30 and 0.60, and a low correlation below 0.30 [17]. Low correlations were expected between those dimensions that are theoretically unrelated constructs, thereby testing the discriminant validity.

Known-groups validity consists of showing that an instrument can differentiate between groups of patients whose health status differs according to the characteristics of the patients' disease, in this case clinician-rated severity of GERD symptoms.

#### Responsiveness to change

Responsiveness to change was assessed using effect size and standardized response mean. The effect size anchors the changes against the variability in the sample, and is calculated by dividing the mean change by the standard deviation at baseline. The standardized response mean preserves the relation to a statistical test, and is calculated by dividing the mean change by the standard deviation of the change. According to Cohen's definition, an effect size ≥ 0.8 indicates a large responsiveness to change [18].

### Statistical methods

Data entry took place in an Oracle-based clinical database. Statistical analyses and computerized data checks were performed using Statistical Analysis System (SAS, version 8.02; Cary, 2001). The QOLRAD questionnaire was analysed as mean score per domain. If data were missing from one or more item, the mean of the completed items in the same domain was used, provided that more than half of the items in that domain had been completed.

## Results

### Demographic and clinical characteristics

A total of 1166 patients were entered in the study (visit 1). Of these, 1033 (88.6%) took part in visit 2 and 957 (82.1%) took part in visit 4. The reasons for drop-out were withdrawal, loss to follow up and failure to fulfil eligibility criteria. The mean age was 49.1 years (standard deviation [SD]: 13.5) at visit 1, 49.3 years (SD: 13.4) at visit 2, and 49.3 years (SD: 13.3) at visit 4. Patient demographics and clinical data are summarized in Table 1.

**Table 1:** Patient demographics and clinical data.

Variables	Visit 1 (N = 1166) %	Visit 2 (N = 1033) %	Visit 4 (N = 957) %
Age (years)			
18-29	9.1	8.6	8.5
30-39	18.8	18.3	18.3
40-49	22.7	22.9	23.0
50-59	27.0	27.5	28.2
≥ 60	22.4	22.7	22.0
Male	53.2	54.4	55.1
Caucasian	97.5	97.8	98.2
Heartburn^a^			
None	0.3	76.5	76.5
Mild	2.5	20.2	18.7
Moderate	67.2	2.5	4.3
Severe	30.1	0.8	0.4
Regurgitation^b^			
None	19.8	84.7	90.6
Mild	24.7	13.0	7.3
Moderate	37.4	1.8	1.8
Severe	18.1	0.5	0.1
Dysphagia^c^			
None	68.7	95.0	96.0
Mild	17.6	4.1	3.4
Moderate	10.4	0.7	0.3
Severe	3.3	0.1	0.0
Satisfaction^d^			
Completely satisfied	-	71.2	79.1
Quite satisfied	-	24.1	15.8
Quite dissatisfied	-	3.9	3.8
Completely dissatisfied	-	0.8	1.0

^a^In the week before the visit; unknown for 0.1% of patients at visit 4.

^b^In the week before the visit; unknown for 0.2% of patients at visit 4.

^c^In the week before the visit; unknown for 0.2% of patients at visits 2 and 4.

^d^In the week before the visit; unknown for 0.3% of patients at visit 4.

All patients had a history of heartburn of at least 3 months, and the majority had episodes of heartburn of at least moderate severity on at least 3 days in the week prior to the study (Table 1). As rated by the investigator at baseline, 97.3% of patients had moderate or severe heartburn, 55.5% had moderate or severe regurgitation, and 13.7% had moderate or severe dysphagia. At visit 2, symptoms of heartburn, regurgitation and dysphagia were mild or absent in 96.7%, 97.7% and 99.1% of patients, respectively. Furthermore, 78.1% of patients reported having symptoms on at most one day a week. At visit 2, 95.3% of patients reported being satisfied with the way their reflux symptoms were treated.

### Psychometric evaluation

#### Reliability

Cronbach's α scores ranged from 0.83 (Vitality) to 0.92 (Emotional distress) at baseline, thus demonstrating internal consistency (Table 2). High ceiling effects (defined as > 30% of patients having the maximum score, i.e. 'none of the time' or 'none at all') were observed in 5 of the 25 items of the QOLRAD questionnaire. Four of these were in the Physical/social functioning domain. They were 'kept you from doing things with your family' (40.1%), 'difficulty socializing with family' (39.1%), 'unable to carry out daily activities' (38.4%) and 'unable to carry out normal physical activities' (34.8%). The fifth item with a high ceiling effect was in the Emotional distress domain: 'discouraged or distressed' (32.7%). No ceiling effects were observed in the remaining 20 items of the QOLRAD questionnaire.

**Table 2:** Cronbach's α for QOLRAD questionnaire domains at visit 1 (baseline).

QOLRAD domains	Cronbach's α*
Emotional distress	0.92
Sleep disturbance	0.91
Food/drink problems	0.87
Physical/social functioning	0.85
Vitality	0.83

*A high α coefficient (≥ 0.70) suggests good internal consistency and reliability [16].

QOLRAD, Quality of Life in Reflux and Dyspepsia.

#### Construct validity

Pearson correlation coefficients were used to assess the convergent validity. There was a negative correlation between the QOLRAD questionnaire and the clinician-assessed GERD symptom variables across all domains (Table 3). The QOLRAD domains of Sleep disturbance, Food/drink problems, Physical/social functioning and Vitality yielded the strongest correlation with clinician-assessed severity of heartburn. The QOLRAD Sleep disturbance domain also correlated with clinician-assessed severity of regurgitation.

**Table 3:** Correlation coefficients between QOLRAD questionnaire domains and reflux symptom variables at visit 1 (baseline).*

	QOLRAD domain				
	
GERD symptomvariable^†^	Emotional distress	Sleep disturbance	Food/drink problems	Physical/social functioning	Vitality
Dysphagia	-0.13	-0.24	-0.22	-0.19	-0.20
Heartburn	-0.22	-0.35	-0.32	-0.32	-0.32
Regurgitation	-0.20	-0.30	-0.28	-0.28	-0.27
Days with heartburn last week	-0.02	-0.01	-0.07	-0.02	-0.05

*A strong correlation was considered to be over 0.60, a moderate correlation between 0.30 and 0.60, and a low correlation below 0.30 [17].

^†^As assessed by the clinician.

GERD, gastroesophageal reflux disease; QOLRAD, Quality of Life in Reflux and Dyspepsia.

**Figure 1:**
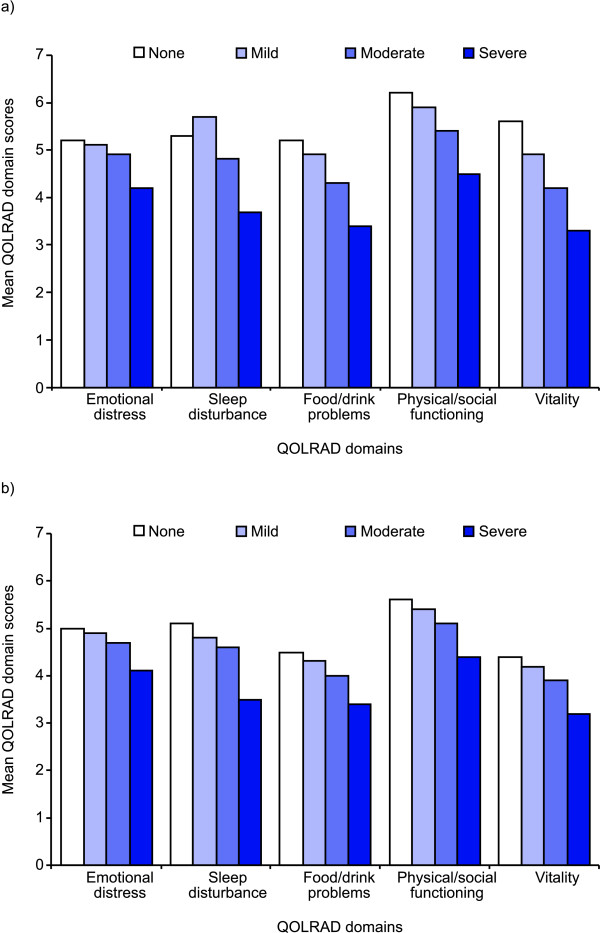
**Quality of Life in Reflux and Dyspepsia (QOLRAD) questionnaire domain scores**. Scores are stratified by clinician-rated severity of a) heartburn and b) regurgitation at baseline (visit 1).

Known-groups validity was used to compare the QOLRAD domain scores with clinician-rated severity of reflux symptoms (Figure 1). All domains of the QOLRAD questionnaire were able to differentiate between groups of patients whose health status differed according to clinician-rated severity of reflux symptoms. Increasing symptom severity was associated with a worsening impact on daily functioning (i.e. a lower QOLRAD score). QOLRAD domain scores negatively correlated with increasing clinician-rated severity of heartburn (Figure 1a) and regurgitation (Figure 1b).

#### Responsiveness to change

Responsiveness to change from visit 1 to visit 2 was evaluated using effect sizes and standardized response means (Table 4). Effect sizes and standardized response means were high (ranging from 1.15 to 1.93 and from 1.17 to 1.86, respectively) indicating a large responsiveness to change [18].

**Table 4:** Effect size and standardized response mean QOLRAD questionnaire domains between visit 1 and visit 2.

QOLRAD domain	Effect size*	Standardized response mean
Emotional distress	1.38	1.45
Sleep disturbance	1.40	1.41
Food/drink problems	1.93	1.86
Physical/social functioning	1.15	1.17
Vitality	1.74	1.76

*An effect size ≥ 0.8 indicates a large responsiveness to change [18].

QOLRAD, Quality of Life in Reflux and Dyspepsia.

#### Mean QOLRAD domain scores

**Figure 2:**
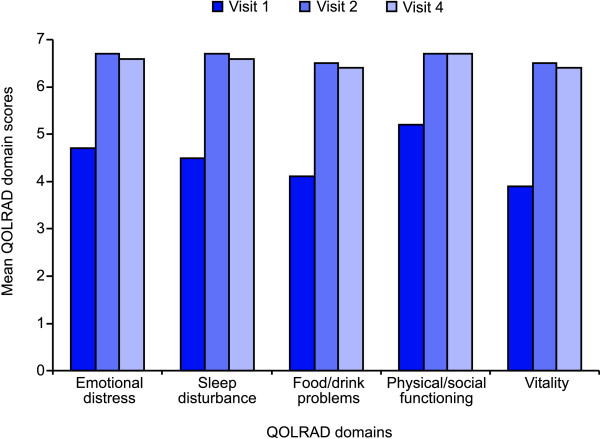
**Mean Quality of Life in Reflux and Dyspepsia (QOLRAD) domain scores**. Results are shown from visit 1 (baseline), visit 2 (after 2, 4 or 8 weeks of acid-suppressive treatment) and visit 4 (after 6 months of acid-suppressive treatment).

Mean QOLRAD domain scores at baseline (visit 1), at visit 2 and at visit 4 are shown in Figure 2. Items were rated on a 7-point Likert scale, with lower values indicating a more severe impact on daily functioning. At baseline, GERD symptoms impacted most strongly on Vitality (mean QOLRAD domain score: 3.9), followed by Food/drink problems (4.1), Sleep disturbance (4.5), Emotional distress (4.7) and Physical/social functioning (5.2). With treatment, mean QOLRAD domain scores increased by between 1.5 points (Physical/Social functioning) and 2.5 points (Vitality), indicating a clinically relevant improvement in patients' daily functioning.

## Discussion

The primary aim of this paper was to establish the psychometric characteristics of the Dutch translation of the QOLRAD questionnaire. The reliability of the translated questionnaire was assessed using internal consistency. All domains of the QOLRAD questionnaire demonstrated internal consistency, with Cronbach's α scores ranging from 0.83 to 0.92. Scores were thus well above the 0.60 required to support construct validity [17]. These results are similar to those obtained for the Italian [12], German [10], Spanish [14], Polish [15] and Afrikaans [19] translations of the QOLRAD questionnaire, for which the overall Cronbach's α scores ranged from 0.77 to 0.95. In the present study, high ceiling effects were observed in five of the 25 QOLRAD domains - four in the Physical/social functioning domain and one in the Emotional distress domain. Sensitivity and responsiveness to change is thus likely to be reduced in these domains.

To assess construct validity, we used convergent validity and known-groups validity. Moderate correlations were found between QOLRAD domains and clinician-assessed severity of heartburn symptoms. Overall, convergent validity was moderate to low, and the highest values were obtained for the heartburn and regurgitation variables, these being the cardinal symptoms of GERD. The higher correlation with heartburn and regurgitation than with dysphagia may reflect that almost all patients had heartburn and regurgitation at study entry, but fewer than one-third had dysphagia. All domains of the QOLRAD questionnaire were able to differentiate between groups of patients whose health status differed according to clinician-rated severity of reflux symptoms, thereby confirming the known-groups validity of the instrument. Known-groups validity was similarly confirmed in the Italian [12], German [10], Spanish [14], Polish [15] and Afrikaans [19] translations of the QOLRAD questionnaire. Furthermore, QOLRAD domain scores negatively correlated with increasing clinician-rated severity of heartburn and regurgitation.

The responsiveness to change of the Dutch QOLRAD questionnaire was tested using effect sizes and standardized response means. According to Cohen's definition, an effect size ≥ 0.8 indicates a large responsiveness to change [18]. Both the effect sizes and the standardized response means of the QOLRAD questionnaire were very high, ranging from 1.15 to 1.93, and from 1.17 to 1.86, respectively. The Dutch translation of the QOLRAD questionnaire thus displayed excellent responsiveness to change.

Reflux symptoms were seen to have a clear and consistently negative impact on patients' lives. QOLRAD scores were lowest in the Vitality domain (mean QOLRAD score: 3.9), indicating that patients were feeling tired or worn out, were generally unwell and had a lack of energy. Scores were also lowest in the Vitality domain in the Italian [12] and Polish [15] translations of the QOLRAD questionnaire (mean scores: 4.8 and 3.8, respectively). Scores were also impaired in the Vitality domain in the German [10], Spanish [14] and Afrikaans [19] translations of the QOLRAD questionnaire (mean scores: 4.4, 4.5 and 3.5, respectively), but were lowest in the Food/drink problems domain in these populations (mean scores: 4.4, 4.5 and 3.5, respectively), indicating that, because of their symptoms, patients were restricted in when or what they could eat and drink.

Virtually all patients reported moderate or severe heartburn in the week prior to the study, and more than half reported moderate or severe regurgitation. At visit 2, symptoms of heartburn and regurgitation were mild or absent in almost all patients. Furthermore, mean QOLRAD domain scores increased by between 1.5 points (Physical/social functioning) and 2.5 points (Vitality). Previous studies have shown that a difference in QOLRAD score of approximately 0.5 points represents a clinically relevant change [4,10]. The improvements in QOLRAD scores observed in the current study thus suggest a clinically relevant improvement in patients' daily functioning with acid-suppressive treatment.

The study has two important limitations. Firstly, test-retest reliability was not reported. Secondly, the study was conducted in gastroenterology centres, and the results are thus particular to patients referred for gastroenterological investigation. Thus, no conclusions can be made as to whether the Dutch translation of the QOLRAD is consistent when measuring a stable variable on two separate occasions, or whether its psychometric characteristics would be equally good in different patient populations with GERD.

## Conclusions

The psychometric characteristics of the Dutch translation of the QOLRAD questionnaire were found to be good, with satisfactory reliability and validity, and excellent responsiveness to change. In addition to the original English-language version, several different language versions of the QOLRAD questionnaire have also been validated [9-12,19]. These, together with the Dutch translation of the QOLRAD questionnaire, provide an excellent basis for collaborative research between different parts of the world, and make international trials more applicable, comparable and generalizable despite differences in language and culture.

## Competing interests

Jonas Carlsson is an employee of AstraZeneca R&D Mölndal. Katarina Halling was employed by AstraZeneca R&D Mölndal at the time the study was conducted.

## Authors' contributions

All authors contributed to the concept and design of the study, to the interpretation of the data and to drafting the manuscript. JC performed the statistical analysis. All authors read and approved the final manuscript.
